# Work Climate Scale in Emergency Services: Abridged Version

**DOI:** 10.3390/ijerph18126495

**Published:** 2021-06-16

**Authors:** José Antonio Lozano-Lozano, Salvador Chacón-Moscoso, Susana Sanduvete-Chaves, Francisco Pablo Holgado-Tello

**Affiliations:** 1 Instituto de Ciencias Biomédicas, Instituto Iberoamericano de Desarrollo Sostenible, Universidad Autónoma de Chile, Santiago 7500912, Chile; 2 Departamento de Psicología Experimental, Facultad de Psicología, Universidad de Sevilla, 41018 Sevilla, Spain; sussancha@us.es; 3 Departamento de Psicología, Universidad Autónoma de Chile, Santiago 7500138, Chile; 4 Departamento de Metodología de las Ciencias del Comportamiento y de la Salud, Universidad Nacional de Educación a Distancia, 28040 Madrid, Spain; pfholgado@psi.uned.es

**Keywords:** work climate, emergency service, abridged version scale, validity, reliability

## Abstract

This study is based on a 40-item work climate scale in hospital emergency services (WCSHES). Teams working in these emergency services experience a heavy workload and have a limited amount of time with each patient. COVID-19 has further complicated these existing issues. Therefore, we believed it would be helpful to draft an abridged version of the 40-item WCSHES, considering both validity and reliability criteria, but giving greater weight to validity. One hundred and twenty-six workers between the ages of 20 to 64 (M = 32.45; standard deviation (SD = 9.73)) years old participated voluntarily in the study. The validity, reliability, and fit model were evaluated in an iterative process. The confirmatory factor analysis yielded appropriate global fit indices in the abridged 24-item version (*Χ*^2^(248) = 367.84; *p* < 0.01, RMSEA = 0.06 with an interval of 90% from 0.05 to 0.07, SRMR = 0.08, GFI = 0.9, AGFI = 0.96, CFI = 0.98, NFI = 0.95, and NNFI = 0.98), along with test criteria validity (*ρ_XY_* = 0.68, *p* < 0.001) and excellent reliability (α = 0.94 and ω = 0.94), maintaining the same conceptualization and usefulness of the original scale. The abridged 24-item version was used to measure four work climate factors (work satisfaction, productivity/achievement of aims, interpersonal relations, and performance at work). Evidence of the usefulness of the new abridged scale is provided along with a description of our study limitations and future areas for development.

## 1. Introduction

The COVID-19 pandemic set off a major health crisis around the world in 2019. Work conditions were riddled with uncertainty, particularly for workers in hospital emergency services, who are subject to a heavy workload, stress, and exhaustion [1].

In organizations providing healthcare, the safety of personnel is a top priority. A positive work climate promotes greater satisfaction, productivity, interpersonal relations, performance [2], and mental wellbeing at work [3]. Work climate is perceived as the quality of the experienced environment that influences the behavior of those employed there and, in the case of healthcare, impacts the effectiveness or quality of patient care [4].

The availability of instruments that assess the quality of the work climate has been a focus of studies in this field. Certain authors have emphasized organizational characteristics [5], psychological features [6] and, in some cases, both [7].

There are validated instruments for both workers in general [8] and specific areas of work, such as professors of dentistry [9], midwives [10], or high school workers [11], yet few instruments have been designed for health services or, more specifically, hospital emergency services. In addition, the elements that comprise work climate vary greatly in each field. When it comes to health, some authors have focused on individual behavior and team interactions [12], safety factors of the organizational climate [13], worker satisfaction and its correlation with absenteeism [14], the productivity of health kiosks [15], and the relationship between work climate and psychological tension among workers [16].

Emergency services in hospitals have specific characteristics that differentiate them from the rest of the hospital services [17]. In most cases, and even more so in the context of the current pandemic, they are a patient’s first line of access to healthcare [18], so they tend to many patients, although not all present extremely serious health situations [19,20].

Emergency department staff are exposed daily to traumatic incidents that require critical split-second decisions [21,22]. They are subjected to higher levels of stress and responsibility than other healthcare professionals [23,24] and are at increased risk of developing post-traumatic stress disorder and depression [25]. Good teamwork skills [21] and interpersonal relationships can act as protective factors for workers’ mental health [26], while improving both job satisfaction [27] and job performance [28]. In this context, a work climate scale that considers the specific characteristics of emergency services is needed.

Within the specific field of emergency services, there are studies that address different constructs related to work climate (Supplementary Material. Table S1. Available at https://osf.io/qmxyk/, accessed on 15 June 2021). These include studies on the following:

- The physical environment [29] and its influence on staff satisfaction and performance. A six-dimension instrument that measures the physical environment: ambience (six items, Cronbach’s α 0.86), user-friendliness (six items, Cronbach’s α 0.76), layout (four items, Cronbach’s α 0.71), amenities (three items, Cronbach’s α 0.62), cleanliness (two items, Cronbach’s α 0.76), and adaptability (three items, Cronbach’s α 0.61), and presents values of internal consistency and dimensionality.
- The safety climate [30,31,32,33,34,35] and other safety-related concepts, such as patient safety culture [36] or safety attitudes [37], understood as all individual and group values that determine the commitment, style, and competence of an organization’s health and safety management.
- Worker relations [38] and its relationship to job satisfaction. This instrument is comprised of three factors, namely: individual worker relations (three items, Cronbach’s α 0.74), supervisor–worker relations (three items, Cronbach’s α 0.79), and organization–worker relations (three items, Cronbach’s α 0.72). Confirmatory factor analysis indicates a good model fit.
- Aspects related to climate, but without information on the psychometric properties of the instruments used, include the following: (a) safety climate and medical errors [39], focused on contextual factors (physical environment, staffing, equipment and supplies, teamwork, nursing, culture, screening and monitoring, information coordination and consultation, and inpatient coordination) and their incidence on any adverse events that can produce medical errors; (b) intrinsic motivation, team climate, and burnout [40], focused on connections between these three constructs; (c) violence prevention climate [41], focused on patient and staff factors that help prevent violence in emergency services; and (d) healthcare climate [42], a test that measures factors related to the hospital nursing climate, nursing unit climate, climate strength, patient safety, and medication safety.
- Not focused on the work team climate, but integrate climate as part of the general characteristics of the organization, such as the organizational climate [43], organizational culture [44], and organizational climate for quality [45]. Only the last of these proposals report on the psychometric properties of the instruments used, referring to their internal consistency, instrument dimensionality, and concurrent validity.
- Work climate [46], which integrates aspects of work groups and the organization, and measures aspects related to job satisfaction, productivity/achievement of aims, interpersonal relations, and performance at work. This scale is the only one developed for emergency services in a Spanish-speaking cultural context that has good psychometric properties (internal consistency study, content validity, construct validity, and concurrent validity). However, the authors have noted the length of the scale as an impediment, considering that emergency department workers receive more patients than they can handle, making it difficult for them to find the time to complete the survey. In addition, eight items from the original scale presented differential item functioning (DIF).

The main objective of this study was to develop an abridged version of the previously validated work climate scale in emergency health services [46], eliminating the biased items detected in the original scale, in order to create a rapid and effective assessment scale of work climate conditions in hospital emergency services (see Table 1).

## 2. Materials and Methods

An instrument validation study was carried out between January 2019 and January 2021.

### 2.1. Participants

All of the employees of the hospital emergency department at the Davila Clinic in Santiago, Chile, with at least one year of seniority were invited to be part of the sample after several invitations and meetings with the service directors. Ultimately, of the 143 hospital emergency service workers meeting the criterion who were asked to participate, 126 (88.11%) agreed. Their ages ranged from 20 to 64 (M = 32.45 and SD = 9.74); 74 (58.7%) were women and 52 (41.3%) were men. Participants hailed from eight Spanish-speaking countries: 114 from Chile (90.5%); 5 from Colombia (4%); 2 from Venezuela (1.6%); and 1 each from Uruguay (0.8%), Spain (0.8%), Bolivia (0.8%), Peru (0.8%), and Cuba (0.8%). Regarding their profession, 44 (34.9%) were emergency technicians, 35 (27.8%) were nurses, 30 (23.8%) were doctors, 9 (7.1%) were service assistants, 6 (4.8%) were administrative personnel, and 2 (1.6%) were security personnel. Their time working in their profession ranged from 1 to 42 years (M = 7.32; SD = 7.40) and their time working at that particular clinic ranged from 1 to 27 years (M = 4.27; SD = 4.59). Table 1 shows a detailed description of the sample, including frequencies and percentages.

### 2.2. Measures

The instruments used were (a) an instruction sheet reporting on the study objectives and use of information, (b) an ad-hoc questionnaire to assess sociodemographic variables, (c) the original scale (a second-order factor model with four factors (RMSEA = 0.079, GFI = 0.97, AGFI = 0.97, CFI = 0.97, NFI = 0.95, and NNFI = 0.97)) with adequate reliability and validity indexes (α = 0.96, ω = 0.96; *ρ_XY_* = 0.68) [46], consisting of 40 five-point Likert-scale items divided into four factors ((F1) work satisfaction, (F2) productivity/achievement of aims, (F3) interpersonal relations, and (F4) performance at work), and (d) an informed consent sheet.

### 2.3. Procedure and Data Analysis

Once the Human Beings Subcommittee of the Scientific Ethics Committee at the Autonomous University of Chile issued the corresponding authorization, as required by the National Commission at the National Agency for Research and Development of Chile and the Technical Directorate of Hospital Emergency Services, a content study was conducted to assess the linguistic adaptation of the work climate scale in emergency health services. Standard forward and backward translations were used, as stipulated by the International Test Commission [47]. The reason for the linguistic adaptation was that the original version had been created and validated in Spain and the abridged version was obtained with data from Chile. As part of the content study, in order to gauge whether the instructions and item contents had similar meanings for all of the intended targets, a team of two local doctors and one nurse (native speakers of the language variant who also administered and responded to the scale), and three bilingual methodologists (who administered the scale) agreed on the instructions and item contents to be modified.

A room with five personal computers was set up, in which the qualifying personnel working for hospital emergency services at the Davila Clinic were invited to participate during their workday. Two researchers were on site two days a week at different times to monitor the information registration process using Google Forms.

In the design of the abridged version scales [48,49], both validity and reliability criteria need to be considered. In addition to evaluating the degree to which a scale gains reliability when items are deleted, the validity of this abridged version depended on both its internal structure and its relationship to the variables of the original scale.

Once the data were gathered, an iterative process was used to reduce the number of items. Firstly, the validity of the scale was assessed based on an analysis of differential item functioning (DIF) obtained from the original scale [46]. A proposal was then made to delete the items that presented DIF according to gender (items 3, 6, 23, 27, and 29), work experience (items 2, 5, and 18), age (items 5, 6, and 18), and the type of working relationship (items 5, 6, and 18) in order to avoid validity threats associated with the degree of generalization of interpretations based on test scores and sample characteristics [50]. Subsequently, reliability coefficients were calculated for the original factors with and without the DIF items. The correlation between the original factors and the factors excluding the DIF items, with the criterion, were calculated as follows: *ρ_XY_*, where X represents the sum of the item scores and Y represents omnibus item 41, “as a whole, the work climate of my work group is good”, of the original scale [46]. Steiger’s Z-test for dependent correlations was used to analyze the correlation differences between the original and abridged versions and the criterion. The representativeness and relevance of the factors was also assessed in order to confirm they were not impacted by the removal of the items. Before eliminating any item, adequate results had to be obtained on the reliability and validity indexes with no significant correlation differences with the original version.

During the iterative process, a review was conducted for each of the four factors (work satisfaction, productivity/achievement of aims, interpersonal relations, and performance at work). The standard deviation (SD) was calculated along with the discrimination rate of each item (ID), the change in reliability if items were discarded (α Cronbach’s alpha and ω McDonald’s omega), and the reliability (RI) and validity (VI) indices of the items. After a relative comparison of the validity and reliability indexes, the most poorly performing items were then removed. Each time an item was removed, the same process was repeated (calculation of reliability coefficients, factor–criterion correlations, and comparison of criterion correlations with the original and the abridged version).

In the last stage, to examine whether the abbreviated scale retained the conceptual framework of the original one, a confirmatory factor analysis (CFA) was carried out for each of the reduced factors using polychoric correlations and unweighted least squares (ULS). Given the ordinal nature and skewness of the measured variables, in addition to the sample size, the ULS method was considered suitable. The advantage of this method is that it does not establish how categorical observed variables are distributed. In contexts like the one of this study, ULS also reduces the likelihood that adequately specified models will be rejected, while increasing the likelihood that poorly specified ones will, thus yielding better results than other estimation methods [51,52]. To assess the global fit of the models, the following indicators were considered: chi-square test (*χ*^2^), accepting the null hypothesis when values *p* ≥ 0.05, which implied a good fit of the model; the root mean square error of approximation (RMSEA), where values lower than 0.05 were considered a good fit, between 0.08 and 0.1 a reasonable fit, and greater than 0.1 unfit [53]; and standardized root mean squared residual (SRMR) model, where values < 0.08 generally indicated an adequate fit [54]. The following fit indicators were interpreted as good if their values were greater than 0.9 [55]: goodness-of-fit index (GFI), the adjusted goodness-of-fit index (AGFI), the comparative fit index (CFI), the normed fit index (NFI), and the non-normed fit index (NNFI). Statistical programs SPSS 26.0 for Windows and LISREL 8.71 [56] were used.

## 3. Results

This iterative review process was carried out to reduce the items in each factor. First, in relation to factor 1 (F1) “satisfaction with work”, items with DIF in the original F1 were eliminated. Reliability indices were calculated for the original 10 items-F1 (alpha 0.87 and omega 0.89) and without the four DIF items (alpha 0.84 and omega 0.87). The correlation of the original 10 items-F1with the criterion was 0.56 and excluding the DIF items, it was 0.44. These correlation differences between the original and abridged F1 and the criterion were not significant (*z* = 1.26, *p* = 0.10). See Table 2.

In stage 1, after excluding the items with DIF, the first item to be eliminated was number 7, after collaboration between the emergency work teams and specialists from other departments. Together, its validity index (0.22) and reliability (0.38) were low. In addition, the alpha and omega remained stable when this item was removed. Therefore, the new F1 was formed by items 1, 4, 8, 9, and 10. The reliability indices for this F1 were alpha 0.87 and omega 0.88. The correlation of this F1 with the criterion was 0.43, while the correlation of the original F1, including the 10 items with the criteria, was 0.56. This difference was not yet significant (*z* = 1.36, *p* = 0.08). Item 8, which considers the relevance of each team member, compensates for the eliminated item. As the validity of the F1 remained stable after eliminating item 7, the reliability remained high, and important information was not missing, another item could be discarded.

In stage 2, according to the validity and reliability indexes, the next candidate for elimination was item 9. Its validity index (0.21) and reliability (0.42) were low. The alpha and omega without item 9 decreased, but still remained high. Therefore, the new F1 was formed by items 1, 4, 8, and 10. The reliability indexes for this new F1 were alpha 0.81 and omega 0.83 and correlation with the criterion was 0.44. The correlation of the original 10 items-F1 with the criteria was 0.56. This difference was not yet significant (*z* = 1.45, *p* = 0.07). It thus became possible to eliminate item 9, which refers to the feeling that work is important, as item 1 covers that information (gives it refers to the feeling that one’s work is important and also having pride in one’s work).

In stage 3, given that the validity of this F1 remained stable, and reliability remained high, another item could be discarded. According to the validity (0.19) and reliability (0.45) indices, the lowest for any item, the next candidate for elimination was item 10. The alpha and omega decreased, but their values remained high. Therefore, the new F1 was comprised of items 1, 4, 8, and 10. The reliability indexes for this new F1 were alpha 0.84 and omega 0.85, and correlation with the criteria was 0.42. The correlation of the original 10 items F1 with the criteria, was 0.56. This difference was not yet significant (z = 1. 26, *p* = 0.10). Item 10 refers to whether the job allows workers to develop their skills and knowledge. The only item that recorded related information was item 5, referring to the experience needed to perform the job well. However, as item 5 presented DIF in the original version, it was dropped. For these reasons, item 10 was maintained.

Moreover, for items 1, 4, and 8, the model did not present adequate global fit indices after a CFA was performed. The fit indices obtained were *χ*^2^(1) = 12.40, *p* ≤ 0.001, RMSEA = 0.30 with a 90% interval of 0.17 to 0.46, SRMR = 0.12, GFI = 0.97, AGFI = 0.82, CFI = 0.92, NFI = 0.92, and NNFI = 0.77. Given these results, with F1 comprised of items 1, 4, 8, and 10, we calculated that the fit indices were *χ*^2^(2) = 8.31, *p* < 0.001, RMSEA = 0.10 with a 90% interval of 0.06 to 0.28, SRMR = 0.05, GFI = 0.99, AGFI = 0.97, CFI = 0.98, NFI = 0.97, and NNFI = 0.93. Therefore, to maintain a reliable F1 and preserve validity (internal structure and criterion validity), the final F1 model should be made up of items 1, 4, 8, and 10 (α = 0.84 and ω = 0.85).

This same iterative process was carried out for each of the factors (all information of the process is accessible in Supplementary Material, Table S2. Available at: https://osf.io/vjstm/, accessed on 15 June 2021). Table 3 shows the specific statistical indices of the items deleted based on DIF and in the iterative evaluation process for each factor.

In factor 2 (F2), for productivity/achievement of aims, items 18, 23, 27, and 29 presented DIF and were thus removed, and subsequently, items 24, 13, 25, and 20 were eliminated considering their validity and reliability indices. The abridged 12-item F2 presented reliability values of α = 0.90 and ω = 0.90. The correlation of the abridged F2 with the criterion was *ρ_XY_* = 0.57, while the correlation of the original 20-item F2 was *ρ_XY_* = 0.62. This difference was not significant (z = 0.61; *p* = 0.27). The 12-item F2 model presented appropriate global fit indices after performing a CFA, with a model fit of *χ*^2^(54) = 131.540, *p* < 0.001, RMSEA = 0.10 with an interval of 90% from 0.07 to 0.13, SRMR = 0.08, GFI = 0.97, AGFI = 0.96, CFI = 0.96, NFI = 0.94, and NNFI = 0.96. The items proposed for elimination did not substantially alter the meaning of F2. Item 13 refers to whether the necessary infrastructure is available, item 24 assesses whether the characteristics of the patient are aligned with the specific service, and item 25 assesses whether the characteristics of the patients that workers see are known. These items could be integrated into item 15, which refers to whether the characteristics of the service are appropriate.

For interpersonal relations, factor 3 (F3), only item 34 could be deleted; an attempt was made to also remove item 35, but the global fit indices of the internal structure were not supported in the model. The reliability rates for the abridged F3 were α = 0.88 and ω = 0.89, and the correlation with the criteria was *ρ_XY_* = 0.79. Although this difference was significant (z = 2.19, *p* = 0.01), the four-item F3 model presented an adequate fit of *χ*^2^(2) = 6.24, *p* = 0.041, RMSEA = 0.10 with an interval of 90% from 0.05 to 0.15, SRMR = 0.04, GFI = 0.99, AGFI = 0.98, CFI = 0.99, NFI = 0.99, and NNFI = 0.97. Item 34, which was eliminated, assesses the quality of personal relationships among team members, but this information could be integrated into item 32, which includes both personal and professional relationships.

In factor 4 (F4), performance at work, it was only possible to remove item 40. An attempt was made to also eliminate item 39, but the fit indices of the internal structure did not support the model. The reliability indices for the resulting scale were α = 0.83 and ω = 0.84 and its correlation with the criteria was *ρ_XY_* = 0.52, while for the original F4, *ρ_XY_* = 0.43, this difference was not significant (z = 0.91; *p* = 0.18). The four-item F4 model presented an adequate model fit of *χ*^2^(2) = 0.29, *p* = 0.87, RMSEA = 0.040 with an interval of 90% from 0 to 0.09, SRMR = 0.00, GFI = 1, AGFI = 1, CFI = 1, NFI = 1, and NNFI = 1. The item deleted (item 40) refers to whether the team is aware of its shortcomings as a group, although these could be recorded individually as part of item 37.

Table 4 lists the statistical indices for each item of each factor, as well as the reliability indices of the resulting abridged scale.

Following the analysis, the proposal for an abridged version of the scale consisted of items 1, 4, 8, and 10 (factor 1, work satisfaction); 11, 12, 14, 15, 16, 17, 19, 21, 22, 26, 28, and 30 (factor 2, productivity/achievement of aims); 31, 32, 33, and 35 (factor 3, interpersonal relations); and 36, 37, 38 and 39 (factor 4, performance at work). In other words, the original 40-item scale was pared down to 24 items. The reliability rates for the abridged version were very satisfactory (α = 0.94 and ω = 0.94) compared with α = 0.96 and ω = 0.96 for the original version. The correlation of the original version with the criteria was *ρ_XY_* = 0.68, and the correlation of the abridged 24-item version with the criteria was also *ρ_XY_* = 0.68, with a model fit of *χ*^2^(248) = 367.84; *p* < 0.01, RMSEA = 0.06 with an interval of 90% from 0.05 to 0.07, SRMR = 0.08, GFI = 0.9, AGFI = 0.96, CFI = 0.98, NFI = 0.95, and NNFI = 0.98.

## 4. Discussion

The abridged version of the work climate scale has no DIF issues and thus boasts a significantly reduced length while maintaining high levels of reliability and validity.

Table 5 shows a comparison of the factor loadings and factor correlation matrix of both versions.

The results show that the abridged version maintains the same factor structure as the long version, without substantially altering the content: factor 1, work satisfaction, reduced by four items, refers to workers’ feelings towards their job and conditions on the job [57]. The items that remain in factor 1 cover the content of the eliminated items. For example, item 1, “we take pride in our work”, includes the information provided by item 9, “our work is important”, as both refer to thoughts and feelings about one’s work. Item 4, “we strive to achieve successful outcomes”, encompasses the information provided by item 2 (“we seek to understand the needs of our clients”), item 3 (“we readily adapt to new circumstances”), and item 6 (“our workday is adequate to develop our work”). Adapting to new circumstances, the workday, and the needs of patients suggests an effort to achieve successful results. Item 8, “relevance of the job of each member”, covers the information in item 7, “good relations with the other services”. By definition, emergency departments are closely interconnected with the other departments of the hospital, as they must treat a range of pathologies from different areas of medicine; additionally, recognizing the relevance of each team member implies good inter-service relations to perform the work well. Finally, item 10, “we develop our skills and knowledge”, covers the information from the deleted item 5, “we have experience to do our work well”, as developing skills and knowledge implies having the necessary experience to perform the job well. Therefore, work satisfaction would comprehend information related to (a) the feeling of pride; (b) the relevance of each professional in his or her job [58]; (c) the effort made to achieve successful results [59], which not only influences job satisfaction [60], but life satisfaction in general [61]; and (d) the development of workers’ skills and knowledge [62]. This factor can be useful for identifying a worker’s job satisfaction level. A high value for this factor would imply that workers are proud of their work, as they consider it relevant, believe they achieve successful outcomes related to patient care, and develop skills and knowledge for professional growth. In contrast, a low score on this factor would imply job dissatisfaction which, together with the burnout and stress inherent to the service [63], would favor the intention to leave the job [64] and even the profession [65].

Factor 2, productivity/achievement of aims, reduced to 12 items, refers to the perception that workers have everything they need to do their job and achieve their objectives [66]. The information recorded in the eight deleted items could be included in the remaining items. For example, item 15 (“the characteristics of our service are appropriate”) could encompass the right characteristics for an emergency service to achieve its objectives and productivity, such as having the necessary resources (item 13, “we have the necessary infrastructure”), critical and urgent health needs on the part of the patients they see (item 24, “our type of patient fits with the service”), or that the team is aware of the characteristics of the patients (item 25, “we know our patients’ characteristics”). Factor productivity/objective achievement would gather information related to (a) the quality of the work performed [67], (b) having a common purpose as a work team [68], (c) the necessary training and education [69], (d) the characteristics of the service [70], (e) the operation of the service [70], (f) productivity [71], (g) recognition of the work performed [72], (h) esteem of peers [73], (i) coordination with other services [74], (j) having a work plan [75], and (k) clarity of work expectations [76]. A high score for this factor would imply that the workers are known for their productivity and the quality of the work they perform, that they have a common purpose and a team plan for achieving the service’s objectives, that they receive the necessary training to perform their work well, that the characteristics of the service are appropriate, that the service functions correctly both internally and in coordination with other hospital services, and that the professionals feel recognized for the work they perform as specialists. Conversely, a low score would indicate that the team is not productive and faces obstacles to achieving its objectives. Therefore, each of the worst-performing aspects could be identified, and improvement measures could be implemented.

Factor 3, interpersonal relations, reduced to four items, refers to workers’ feelings when interacting with the team [8]. The only item eliminated was item 34, “I have good personal relationships”, but its content could be incorporated in item 32, “good relationship between members”, as this deals with both personal and professional relationships. This factor would allow for information to be gathered on (a) intragroup communication [77], (b) the quality of intragroup relations [78], (c) the feeling of comfort on the team, and (d) the feeling of working in a good intragroup climate [79]. A high score for this factor would indicate that the workers have a good relationship, that the necessary spaces are established for intra-group communication, and that the professionals feel comfortable working with the team and value it positively. On the contrary, a low score would imply interpersonal problems, thus serving to identify those aspects that could be improved as a team.

Finally, factor 4, performance at work, reduced to fours items, refers to all aspects related to worker integration on the job [2,80]. Although item 40 on shortcomings as a group is not included in the abridged scale, its information could be recorded individually for each of the work team members through item 37, “I know my professional shortcomings”. Therefore, the performance at work factor would make it possible to record information related to (a) team capabilities, (b) professional limitations and deficiencies [81], (c) team functioning, and (d) the quality of patient screening with respect to the service the team provides [82]. A high score here would imply that the team has well-defined functions and is aware of its capabilities, that they know their limitations, and that the service is prepared for the type of pathology the patient suffers. On the other hand, a low score for this factor would imply that the work performance is not adequate, either because people’s roles are not clear, because the team’s capabilities and limitations are not considered, or because the profile of the patient being seen does not correspond to the specialty of the service. Identifying each of these aspects could help improve them.

The abridged scale maintains novel elements related to the coordination with the other hospital services, the assessment of the weaknesses of each team worker, the limits on each worker’s tasks based on his/her area of expertise, and the recognition of the work carried out. In addition, both the original scale and the abridged version are the only ones created and validated specifically for hospital emergency services. It should be noted that the use of validated scales has been found to enhance the quality of intervention programs [83].

Table 6 presents a description of the response values for each item and factor in the abridged version.

The abridged version (24 items) allows for the work climate at hospital emergency services to be evaluated globally and for each individual worker. Besides being used to assess evaluate the global work climate in hospital emergency services, it can be used for each of the factors or items to detect areas for work climate improvement. For example, if that the scale reveals that 28.5% of the team is not receiving the necessary training to do their work effectively, the team manager can organize training according to the team’s needs. Similarly, it can be used to assess effectiveness; for example, if the scale shows that 50.1% of the team feels that their work is not recognized, activities to improve recognition can be introduced before using the scale for a new assessment, allowing for the use of inferential statistics to measure the impact of the activities introduced in this regard.

The abridged scale proposed herein can be a simple way to take actions that improve the quality of the work climate and prevent exhaustion at work in hospital emergency services, enhancing the satisfaction, productivity, interpersonal relations, and performance of workers [84].

One of the limitations of this study is the sample size, although it is representative of the specific hospital emergency service being evaluated. Our procedure consisted of collecting data for two years at one of Chile’s largest hospitals. We initially planned to apply the instrument to other hospital emergency services, but because of the social unrest in Chile in late 2018, followed by the COVID-19 outbreak in early 2019, it became impossible. Nevertheless, given that the model is robust and the structure is clear [85,86], a sample of over 100 subjects should suffice. In our case, the fit indices were completely satisfactory for each of the factors and for the overall model. Another limitation is the use of an indicator for the study of the criterion validity. However, some guidelines for improving and reporting the psychometric soundness of the instruments include one that relates the attributes of an instrument to performance on a criterion [87]. The question of the generalizability of the instrument could be a limit of this study, as the sample was confined to workers at a single hospital emergency service in Chile. Another limitation could be the elimination of potentially relevant items because of DIF, such as item 29, “we participate in the decisions of our group”.

Once the pandemic has subsided, we intend to access a larger sample and apply this and other instruments that measure the same construct at other hospital emergency services in order to analyze the convergence and discrimination with other instruments. An additional goal of further studies will be examining the factorial invariance of the proposed structural model to determine whether the scale can be extended to different hospitals, countries, genders, and professional categories. In future applications of the scale, we are going to include an explicit question about the respondent’s level of participation in work team decisions as part of the final open-ended question of the scale. After analyzing the contents of the responses to the open item, we will evaluate the possibility of including other items on work team decision-making or enhance/add other aspects not included on the scale. Additionally, the wording of the items (such as the use of “I” instead of “we”) will be analyzed further to detect any method effects.

## 5. Conclusions

In conclusion, the proposed scale gathers all of the contents of the original version and maintains the utility, validity, and reliability criteria, but its abridged version facilitates data collection by reducing the time needed to complete it. It also eliminates those items that presented DIF in the original version of the scale. The final printable versions (English and Spanish) are ready to be used as Supplementary Materials; Tables S3 and S4. They are available at https://osf.io/jy7gf/ (accessed on 15 June 2021).

## Figures and Tables

**Table 1:** Sociodemographic variables of emergency services.

Variables		N (%)
Gender	Women	52 (41.3)
	Men	74 (58.7)
Age	18–40	10 (81.0)
	41–60	23 (18.3)
	>60	1 (0.8)
Profession	Emergency technicians	44 (34.9)
	Nurses	35 (27.8)
	Doctors	30 (23.8)
	Service assistants	9 (7.1)
	Administrative personnel	6 (4.8)
	Security personnel	2 (1.6)
Type of employment contract	Indefinite contract	102 (81.0)
	Training contract	9 (7.1)
	Temporary contract (part-time)	8 (6.3)
	Temporary contract	7 (5.6)

**Table 2:** Descriptive analysis of items presenting DIF eliminated during the iterative review process for factor 1—work satisfaction.

Iterative Review Process	Item	SD	ID	α	ω	RI	VI
Stage 1	1	0.80	0.62	0.85	0.88	0.49	0.28
	2 *	0.71	0.69	0.85	0.87	0.49	0.21
	3 *	0.74	0.60	0.86	0.88	0.45	0.24
	4	0.68	0.79	0.84	0.87	0.54	0.26
	5 *	0.82	0.51	0.86	0.89	0.42	0.22
	6 *	1.04	0.40	0.88	0.89	0.41	0.36
	7 **	0.94	0.40	0.87	0.89	0.38	0.22
	8	0.75	0.73	0.85	0.87	0.55	0.30
	9	0.56	0.69	0.85	0.87	0.39	0.21
	10	0.71	0.67	0.85	0.88	0.48	0.19
Stage 2	1	0.80	0.63	0.82	0.85	0.50	0.28
	4	0.68	0.80	0.79	0.82	0.55	0.26
	8	0.75	0.70	0.79	0.83	0.52	0.30
	9 **	0.56	0.75	0.80	0.83	0.42	0.21
	10	0.71	0.67	0.80	0.84	0.47	0.19
Stage 3	1	0.80	0.60	0.84	0.84	0.48	0.28
	4	0.68	0.79	0.75	0.76	0.54	0.26
	8	0.75	0.68	0.80	0.81	0.51	0.30
	10	0.71	0.64	0.81	0.83	0.45	0.19

SD—standard deviation; ID—item discrimination; α—Cronbach’s alpha if the item is deleted; ω—McDonald’s omega if the item is deleted; RI—reliability index; VI—validity index; *—removed DIF items; **—items proposed to be removed.

**Table 3:** Descriptive analysis of items presenting DIF eliminated during the iterative process.

Factor	Item	SD	ID	α If the Item Is Deleted	ω If the Item Is Deleted	RI	VI
Factor 1: Work satisfaction (α = 0.88; ω = 0.89)	1	0.80	0.62	0.85	0.88	0.49	0.28
	2 *	0.71	0.69	0.85	0.87	0.49	0.21
	3 *	0.74	0.60	0.86	0.88	0.45	0.24
	4	0.68	0.79	0.84	0.87	0.54	0.26
	5 *	0.82	0.51	0.86	0.89	0.42	0.22
	6 *	1.04	0.40	0.88	0.89	0.41	0.36
	7 **	0.94	0.40	0.87	0.89	0.38	0.22
	8	0.75	0.73	0.85	0.87	0.55	0.30
	9 **	0.56	0.69	0.85	0.87	0.39	0.21
	10	0.71	0.67	0.85	0.88	0.48	0.19
Factor 2: Productivity/achievement of aims (α = 0.93; ω = 0.93)	11	0.84	0.67	0.92	0.92	0.36	0.57
	12	0.82	0.64	0.92	0.92	0.27	0.52
	13 **	0.87	0.48	0.93	0.92	0.18	0.42
	14	0.98	0.56	0.93	0.92	0.21	0.55
	15	0.92	0.63	0.93	0.92	0.31	0.58
	16	0.75	0.72	0.92	0.92	0.35	0.54
	17	0.83	0.68	0.92	0.92	0.34	0.57
	18 *	0.92	0.68	0.92	0.92	0.46	0.63
	19	1.12	0.65	0.93	0.92	0.63	0.73
	20 **	0.93	0.50	0.93	0.92	0.35	0.47
	21	1.10	0.64	0.93	0.92	0.56	0.70
	22	1.01	0.52	0.93	0.92	0.32	0.53
	23 *	0.93	0.70	0.92	0.92	0.47	0.65
	24 **	1.01	0.37	0.93	0.93	0.20	0.38
	25 **	0.78	0.55	0.93	0.92	0.31	0.43
	26	0.74	0.65	0.93	0.92	0.32	0.49
	27 *	1.07	0.61	0.93	0.92	0.59	0.66
	28	0.81	0.67	0.92	0.92	0.39	0.54
	29 *	1.15	0.54	0.93	0.92	0.47	0.62
	30	0.82	0.60	0.93	0.92	0.30	0.49
Factor 3: Interpersonal relations (α = 0.89; ω = 0.89)	31	0.83	0.77	0.86	0.86	0.57	0.64
	32	0.80	0.78	0.86	0.86	0.57	0.63
	33	0.73	0.76	0.87	0.86	0.47	0.56
	34 **	0.72	0.64	0.89	0.90	0.40	0.47
	35	0.77	0.73	0.87	0.87	0.55	0.56
Factor 4: Performance at work (α = 0.84; ω = 0.84)	36	0.85	0.70	0.80	0.79	0.48	0.59
	37	0.72	0.63	0.82	0.81	0.27	0.45
	38	0.78	0.73	0.79	0.79	0.39	0.56
	39	0.87	0.62	0.82	0.82	0.22	0.54
	40 **	0.69	0.56	0.84	0.83	0.22	0.38

SD—standard deviation; ID—item discrimination; α—Cronbach’s alpha if the item is deleted; ω—McDonald’s omega if the item is deleted; RI—reliability index; VI—validity index; *—items presenting DIF; **—items proposed to be removed.

**Table 4:** Descriptive statistical indices for the items of each factor.

Factor	Item	SD	ID	α If the Item Is Deleted	ω If the Item Is Deleted	RI	VI
Factor 1: Work satisfaction (α = 0.84; ω = 0.85)	1	0.80	0.56	0.84	0.84	0.44	0.28
	4	0.68	0.68	0.75	0.76	0.46	0.26
	8	0.75	0.68	0.80	0.81	0.50	0.30
	10	0.71	0.59	0.82	0.83	0.42	0.19
Factor 2: Productivity/achievement of aims (α = 0.90; ω = 0.90)	11	0.84	0.72	0.89	0.89	0.61	0.36
	12	0.82	0.65	0.89	0.89	0.54	0.27
	14	0.98	0.52	0.90	0.90	0.51	0.21
	15	0.92	0.60	0.89	0.89	0.55	0.31
	16	0.75	0.71	0.89	0.89	0.53	0.35
	17	0.83	0.67	0.89	0.89	0.55	0.34
	19	1.12	0.55	0.90	0.90	0.62	0.63
	21	1.10	0.52	0.90	0.90	0.57	0.56
	22	1.01	0.46	0.90	0.90	0.46	0.32
	26	0.75	0.68	0.89	0.90	0.51	0.32
	28	0.81	0.68	0.89	0.89	0.55	0.39
	30	0.82	0.65	0.90	0.90	0.54	0.30
Factor 3: Interpersonal relations (α = 0.88; ω = 0.89)	31	0.84	0.57	0.85	0.85	0.48	0.57
	32	0.80	0.52	0.84	0.84	0.42	0.57
	33	0.73	0.57	0.87	0.87	0.42	0.48
	35	0.77	0.63	0.87	0.88	0.49	0.55
Factor 4: Performance at work (α = 0.8; ω = 0.84)	36	0.85	0.73	0.77	0.78	0.62	0.48
	37	0.72	0.57	0.81	0.82	0.41	0.27
	38	0.78	0.67	0.74	0.74	0.52	0.39
	39	0.87	0.50	0.83	0.84	0.44	0.22

SD—standard deviation; ID—item discrimination, α—Cronbach’s alpha if the item is deleted, ω—McDonald’s omega if the item is deleted, RI—reliability index, VI—validity index.

**Table 5:** Comparison of factor loadings and factor correlation matrix of both scale versions.

	Original Version (40 Items)				Abridged Version (24 Items)			
Items	F1	F2	F3	F4	F1	F2	F3	F4
1. We take pride in our work	0.69				0.68			
2. We seek to understand the needs of our clients	0.64							
3. We readily adapt to new circumstances	0.51							
4. We strive to achieve successful outcomes	0.71				0.84			
5. We have experience to do our work well	0.40							
6. Our workday is adequate to develop our work	0.68							
7. Good relations with the other services	0.87							
8. Relevance of the job of each member	0.73				0.85			
9. Our work is important	0.73							
10. We develop our skills and knowledge	0.69				0.76			
11. Our work group is known for quality work		0.64				0.82		
12. We have a common purpose		0.75				0.76		
13. We have the necessary infrastructure		0.75						
14. We receive the necessary training		0.74				0.60		
15. The characteristics of our service are appropriate		0.76				069		
16. Our service works correctly		0.76				0.80		
17. Our work group is known for its productivity		0.64				0.77		
18. We feel motivated doing our work		0.71						
19. The merit of our good job is recognized		0.73				0.61		
20. Our colleagues value our profession		0.77						
21. We are appreciated for the work we do		0.87				0.58		
22. Our specialization is recognized		0.73				0.52		
23. Our expectations have been fulfilled		0.79						
24. Our type of patient fits with the service		0.65						
25. We know our patients’ characteristics		0.79						
26. We coordinate with the other hospital services		0.55				0.78		
27. We are recognized for our individual contributions		0.81						
28. We have a plan that guides our activities		0.72				0.78		
29. We participate in the decisions of our group		0.82						
30. We know what is expected in our work		0.71				0.75		
31. We have good communication within the group			0.86				0.87	
32. Good relationship between members			0.81				0.79	
33. I feel comfortable with my work group			0.87				0.85	
34. I have good personal relationships			0.84					
35. We work in a good work group climate			0.90				0.95	
36. We understand each other’s capabilities				0.64				0.92
37. I know my professional shortcomings				0.64				0.72
38. We know the functions of the members				0.67				0.84
39. Our patients fit the specialty of our service				0.68				0.68
40. We know our shortcomings as a group				0.55				
F2	0.73				0.88			
F3	0.71	0.74			0.51	0.60		
F4	0.81	0.70	0.61		0.83	0.75	0.62	

**Table 6:** Response values for each item and factor (abridged version).

	Score Assigned on the Scale (%)						
	1	2	3	4	5	M	SD
Factor 1. Work Satisfaction	1.4	0.4	5.4	22.0	70.8	4.60	0.73
1. We take pride in our work	1.6	0.8	7.1	22.2	68.2	4.55	0.80
2. We strive to achieve successful outcomes	1.6	0.0	2.4	23.0	73.0	4.66	0.68
3. Relevance of the job of each member	1.6	0.0	6.3	19.0	73.0	4.62	0.75
4. We develop our skills and knowledge	0.8	0.8	5.6	23.8	69.0	4.60	0.71
Factor 2. Productivity/Achievement of aims	1.9	5.0	17.9	38.9	36.2	4.02	0.96
5. Our work group is known for quality work	1.6	1.6	11.9	41.3	43.7	4.24	0.84
6. We have a common purpose	0.8	1.6	11.9	27.0	58.7	4.41	0.82
7. We receive the necessary training	0.8	8.7	19.0	36.5	34.9	3.96	0.98
8. The characteristics of our service are appropriate	1.6	3.2	16.7	36.5	42.1	4.14	0.92
9. Our service works correctly	0.8	1.6	20.6	55.6	21.4	3.95	0.75
10. Our work group is known for its productivity	1.6	0.8	16.7	44.4	36.5	4.13	0.83
11. The merit of our good job is recognized	5.6	17.5	27.0	33.3	16.7	3.38	1.12
12. We are appreciated for the work we do	4.0	15.9	27.8	32.5	19.8	3.48	1.10
13. Our specialization is recognized	4.0	5.6	25.4	41.3	23.8	3.75	1.01
14. We coordinate with the other hospital services	0.8	0.8	9.5	43.7	45.2	4.32	0.74
15. We have a plan that guides our activities	0.8	1.6	15.9	42.9	38.9	4.17	0.81
16. We know what is expected in our work	0.8	1.6	12.7	31.7	53.2	4.35	0.82
Factor 3. Interpersonal relationships	1.0	1.0	12.1	41.5	44.4	4.27	0.79
17. We have good communication within the group	0.8	1.6	16.7	37.3	43.7	4.21	0.83
18. Good relationship between members	0.8	1.6	15.1	43.7	38.9	4.18	0.80
19. I feel comfortable with my work group	0.8	0.8	7.9	42.1	48.4	4.37	0.73
20. We work in a good work group climate	1.6	0.0	8.7	42.9	46.8	4.33	0.77
Factor 4. Performance at work	1.4	2.0	8.1	35.7	52.8	4.36	0.82
21. We understand each other’s capabilities	1.6	2.4	8.7	39.7	47.6	4.29	0.85
22. I know my professional shortcomings	0.8	1.6	4.0	31.0	62.7	4.53	0.72
23. We know the functions of the members	1.6	1.6	3.2	28.6	65.1	4.54	7.78
24. Our patients fit the specialty of our service	1.6	2.4	16.7	43.7	35.7	4.10	0.87

## Data Availability

Database Work Climate Scale in Emergency Services Abridged Version available at: https://osf.io/hxz6g/ (accessed on 15 June 2021).

## Supplementary Materials

Table S1. Instruments Related to Work Climate Applied in Emergency Services. Available at: https://osf.io/qmxyk/ (accessed on 15 June 2021); Table S2. Iterative Process of Item Reduction by Factors. Available at: https://osf.io/vjstm/ (accessed on 15 June 2021); Table S3 (English language version) and Table S4 (Spanish language version). Work Climate Scale in Emergency Services: Abridged Version. Final printable version ready to be used. Available at: https://osf.io/jy7gf/ (accessed on 15 June 2021).
